# Comparative efficacy and safety of bone-modifying agents for the treatment of bone metastases in patients with advanced renal cell carcinoma: a systematic review and meta-analysis

**DOI:** 10.18632/oncotarget.20323

**Published:** 2017-08-18

**Authors:** Kenji Omae, Yasushi Tsujimoto, Michitaka Honda, Tsunenori Kondo, Kazunari Tanabe, Shunichi Fukuhara, Toshi A. Furukawa

**Affiliations:** ^1^ Department of Healthcare Epidemiology, Kyoto University Graduate School of Medicine/School of Public Health, Yoshida Konoe-cho, Sakyo-ku, Kyoto 606-8501, Japan; ^2^ Center for Innovative Research for Communities and Clinical Excellence, Fukushima Medical University, Fukushima 960-1295, Japan; ^3^ Department of Urology, Tokyo Women's Medical University, Tokyo 162-8666, Japan; ^4^ Department of Surgery, Southern TOHOKU Research Institute for Neuroscience, Southern TOHOKU General Hospital, 7-115 Yatsuyamada, Koriyama, Fukushima 963-8052, Japan; ^5^ Department of Health Promotion and Human Behavior, Kyoto University Graduate School of Medicine/School of Public Health, Yoshida Konoe-cho, Sakyo-ku, Kyoto 606-8501, Japan

**Keywords:** bisphosphonate, bone-modifying agent, denosumab, renal cell carcinoma, zoledronate

## Abstract

**Objective:**

To determine the comparative efficacy and safety of all available bone-modifying agents (BMAs) for the treatment of bone metastases in patients with advanced renal cell carcinoma (RCC).

**Results:**

Three studies (259 patients) were identified for the systematic review. Two studies that compared zoledronic acid with placebo or no zoledronic acid showed that zoledronic acid reduced the risk of skeletal-related events (SREs) by 68% (pooled hazard ratio [HR]: 0.32; 95% confidence interval [CI]: 0.19–0.55). The quality of evidence was moderate. The incidence of serious adverse events (AEs) was identical (80%) for both treatment arms in one study and not reported in the other study. In the third study that compared denosumab and zoledronic acid among patients with solid tumors or myeloma, a post-hoc subgroup analysis with individual patient data (155 patients) showed an HR of 0.71 (95% CI: 0.43–1.17) for SREs and a risk ratio of 0.86 (95% CI: 0.68–1.08) for serious AEs for denosumab compared to zoledronic acid.

**Materials and Methods:**

We searched the MEDLINE database, Cochrane Library, WHO International Clinical Trials Registry Platform, and ClinicalTrials.gov database up to January 2017 without language restriction. Only randomized controlled trials were included. When relevant data were missing, we contacted the investigators of the original study. The Grading of Recommendation Assessment, Development, and Evaluation approach was used to assess the evidence certainty.

**Conclusions:**

Our moderate-quality evidence indicates that zoledronic acid significantly reduces SREs risk among patients with bone metastases of RCC.

## INTRODUCTION

Bone is the second-most prevalent site of metastases in patients with advanced renal cell carcinoma (RCC) [1]. Approximately one-third of all patients with advanced RCC develop bone metastases [1, 2]. Most bone metastases from RCC are predominantly osteolytic; approximately one-third of patients with bone metastases from RCC develop hypercalcemia, half of them develop long-bone fractures, and the majority need palliative radiation for pain [1]. Any skeletal-related event (SRE), including pain requiring radiation, pathologic fractures, spinal cord compression, bone surgery, and hypercalcemia, place great burden on both the patient and the caregiver by limiting the patient's activities of daily living, decreasing the quality of life (QOL), and increasing medical expenses. Furthermore, the presence of bone metastases may be associated with poor survival in patients with advanced RCC [3].

Randomized controlled trials (RCTs) have shown that in multiple myeloma, breast cancer, and prostate cancer, bone-modifying agents (BMAs) reduce bone pain, improve QOL, and reduce the number of and time to SREs [4]. For bladder cancer, a small RCT including 40 patients with bone metastases from bladder cancer demonstrated a significant reduction in the risk of developing a bone-related complication and an improvement in both the pain score and overall survival (OS) in patients receiving zoledronic acid [5]. Furthermore, the incidence of side effects in the zoledronic acid arm did not increase in comparison to the placebo arm. With respect to RCC, an early subgroup analysis of 46 patients with RCC enrolled in a phase III trial suggested a reduction in SREs and a trend towards improved OS with zoledronic acid compared to the placebo [6]. However, this finding was contested by a post-hoc analysis of 2,749 patients with RCC with bone metastases who were treated in 8 phase II and phase III trials of various targeted agents. This analysis compared patients who were concomitantly treated with bisphosphonate and those who were not, and found that bisphosphonate treatment was not associated with improved progression-free survival (PFS) or OS [3]. However, since this is essentially an observational study, it may be confounded by various factors unrelated to the use of bisphosphonate.

The current European Association of Urology (EAU) guidelines do not provide any recommendation for the treatment of bone metastases with BMAs in patients with RCC [7], but recommend BMAs for patients with bone metastases from prostate cancer and bladder cancer [8, 9]. To the best of our knowledge, no systematic review has thus far focused on this topic. Therefore, the aim of this systematic review was to identify, describe, and summarize randomized evidence regarding the comparative efficacy and safety of all available BMAs for the treatment of bone metastases in patients with RCC.

## RESULTS

### Quantity of evidence obtained

**Figure 1:**
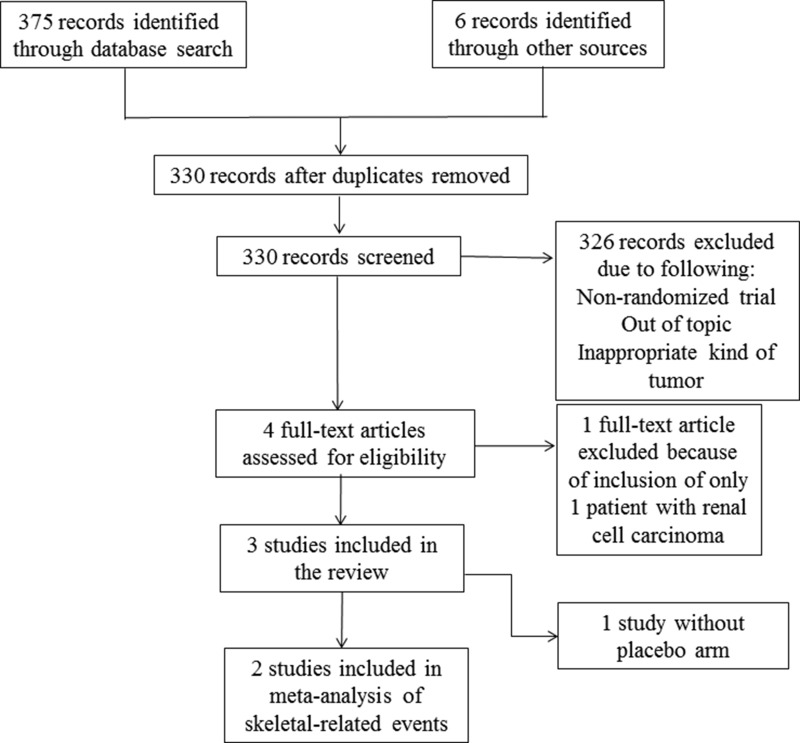
PRISMA flow chart

In total, 330 articles were identified by the literature search. Of these, four studies were eligible for full-text screening. Three studies met the inclusion criteria and were included for evidence synthesis. Figure 1 presents the Preferred Reporting Items for Systematic Reviews and Meta-analyses (PRISMA) flow diagram [13], outlining the study-selection process.

### Characteristics of the included studies

The baseline characteristics of all included studies and an excluded study (with only 1 patient with RCC) are outlined in Table 1. Of the three studies (259 patients) included, one study compared zoledronic acid versus placebo in the treatment of bone metastases in patients with lung cancer and other solid tumors [14]; the study by Lipton et al. [6] included 2 zoledronic acid arms of different doses (4 mg versus 8/4 mg per 3 weeks) and presented a post-hoc analysis of the RCC subgroup. However, we only used data of the 4-mg zoledronic acid arm because the 8-mg dose is not currently approved for use. Another study by Broom et al. [15] compared zoledronic acid in combination with everolimus versus everolimus alone in patients with RCC with bone metastases. The third study compared denosumab versus zoledronic acid for the treatment of bone metastases in patients with advanced cancer (excluding breast and prostate cancer) or multiple myeloma, in which only SREs were reported from subgroup analysis of patients with RCC [16, 17]. Therefore, we conducted an unplanned subgroup analysis using unpublished individual patient data (IPD) of patients with RCC (155 patients) from the study by Henry et al. (1,776 patients) [16], which was kindly shared through Amgen. In all the studies, patients with severe renal impairment were excluded.

**Table 1:** Main characteristics of eligible studies and an excluded study (with only 1 patient with renal cell carcinoma)

Author	Year	Original trial	Design	Uni-/multicenter study	No. of patients with renal cell carcinoma included					Study end points	Ethnicity	Study details
					Total	Zoledronic acid	Denosumab	Clodronate	Placebo or no bone-modifying agent			
Broom RJ et al. [15]	2015	-	Randomized phase 2	Multicenter	30	15			15	Urine N-telopeptide level, plasma C-telopeptide, quality of life, progression-free survival, overall survival, response rate, skeletal-related events, adverse events	European, Maori, Pacific Islander, Asian	Study was designed to evaluate the effects of the addition of zoledronic acid to everolimus treatment
Henry D et al. [16]	2014	Henry et al. 2011	Subgroup phase 3	Multicenter	155	85	70			Skeletal-related events, adverse events, urine N-telopeptide, bone-specific alkaline phosphatase, quality of life, progression-free survival, overall survival	White or Caucasian, Black or African American, Hispanic or Latino, Asian	Individual patient data shared through Amgen was analyzed
Lipton A et al. [6]	2003	Rosen et al. 2004	Subgroup phase 3	Multicenter	74	55			19	Skeletal-related events, adverse events, response rate, overall survival	Unclear	Patients in the 8/4 mg zoledronic acid arm were excluded from analyses (*n* = 28)
Vinholes et al. [19]	1997	-	Randomized, double-blind trial	Unicenter	1			1		Urinary calcium, hydroxyproline, deoxypyridinoline, pyridinoline, N-telopeptide, C-telopeptide, free deoxypyridinoline	Unclear	This study was excluded from review (*n* = 1)

### RoB assessment of the included studies on SREs

**Figure 2:**
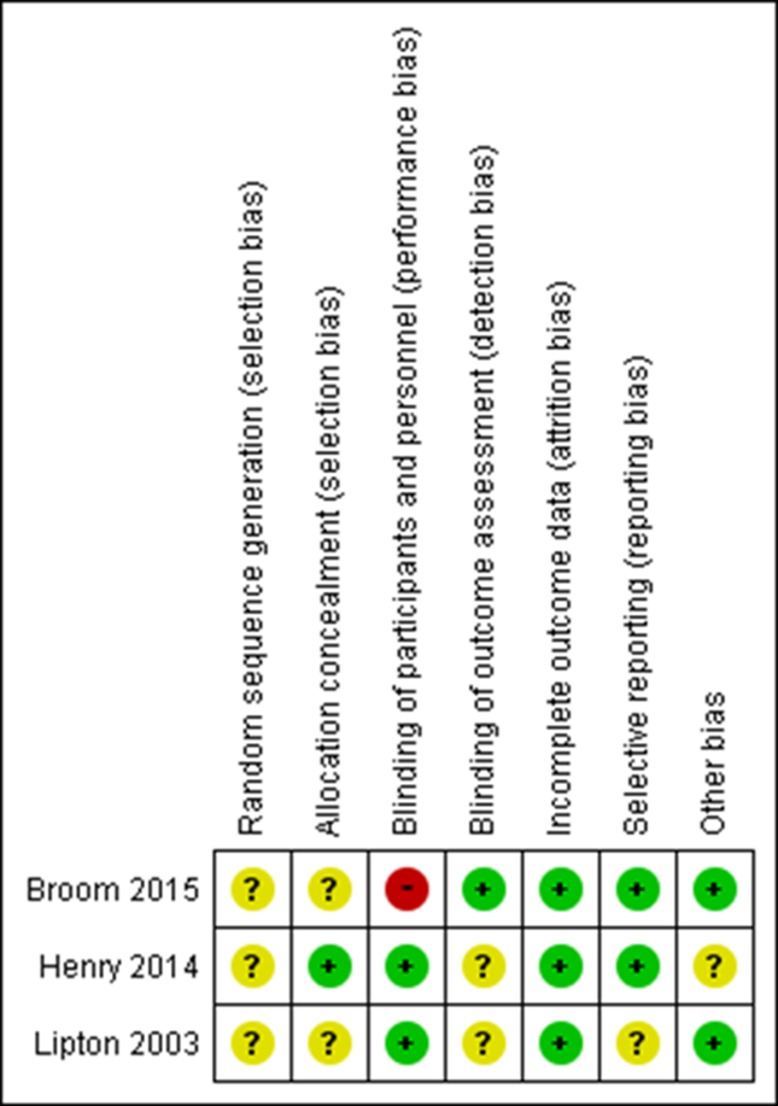
Risk of bias table with the three studies included in the systematic review

Figure 2 summarizes the assessment of RoB for individual studies on SREs. No studies provided details of their random sequence generation. One study was at high risk of bias for blinding of participants. Only one study was explicit about each allocation concealment and blinding of the outcome assessment.

### Effect of BMAs on primary outcomes

The main study outcomes of all included studies are outlined in Supplementary Table 1.

### SREs

**Figure 3:**
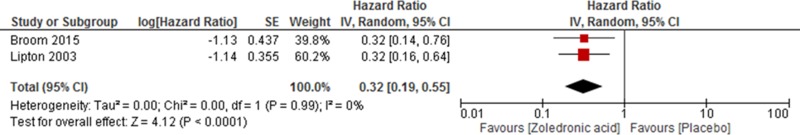
Forest plot and meta-analysis assessing the effect of zoledronic acid on skeletal-related events in renal cell carcinoma patients with bone metastases

The random-effects pooled HR (95% CI) was 0.32 (0.19–0.55), indicating a 68% reduction in the risk of SREs associated with zoledronic acid treatment (Figure 3). No statistically significant heterogeneity was observed amongst the studies (*I*^2^ = 0%). The quality of evidence for SREs was rated “moderate” according the GRADE approach (one level was reduced due to serious study limitations shown in Figure 2). The subgroup analysis of the study that compared denosumab and zoledronic acid did not show a statistically significant risk reduction of SREs with denosumab (HR 0.71; 95% CI 0.43–1.17), but the point estimate was similar with that reported in the overall study [17].

### Serious AEs

No serious osteonecrosis was reported in studies by both Lipton et al. [6] and Broom et al. [15]. In the study by Broom et al., the incidence of serious AEs was identical (80%) for both zoledronic acid plus everolimus and everolimus alone, although 46.7% among patients receiving zoledronic acid plus everolimus stopped zoledronic acid before stopping everolimus: 13.3% owing to symptomatic hypocalcemia, 6.7% owing to persistent asymptomatic hypocalcemia, and 33.3% owing to reduced creatinine clearance. The subgroup analysis of the study by Henry et al. [17], in which the dosage modification of zoledronic acid was allowed based on creatinine clearance, demonstrated a lower rate of serious AEs with denosumab (risk ratio: 0.86; 95% CI: 0.68–1.08), but the difference in the two groups was not significant. Serious osteonecrosis, hypocalcemia, and renal dysfunction were uncommon with both denosumab and zoledronic acid (0% versus 3.5%, 1.4% versus 1.2%, and 2.9% versus 0%, respectively).

### Effect of BMAs on secondary outcomes

### Time to first SRE

Zoledronic acid significantly delayed the time to first SRE compared to controls. The median time to first SRE for zoledronic acid and placebo arms was “not reached” and 2.4 months (*P* = 0.006), respectively, whereas that for zoledronic acid plus everolimus and everolimus alone was 9.6 and 5.2 months (*P* = 0.009), respectively. In the subgroup analysis of Henry et al. [17], although the median time to the first SRE for denosumab was longer than that for zoledronic acid (“not reached” and 11.6 months, respectively), the difference was not statistically significant between the two groups (*P* = 0.16).

### SMR

Only the study by Lipton et al. [6] reported SMR data, in which zoledronic acid significantly reduced SMR by 21% compared to placebo (*P* = 0.014).

### Overall AEs

Osteonecrosis was not observed in the studies by Lipton et al. [6] and Broom et al. [15], although the number of patients included in both studies was small. In the subgroup analysis of the study by Henry et al. [17], the rate of osteonecrosis for denosumab and zoledronic acid was 2.9% and 4.7%, respectively. The study by Lipton et al. [6], in which all patients received daily supplementation with calcium and vitamin D, demonstrated that the rate of hypocalcemia was 19% with zoledronic acid; however, they did not report dosage modification of zoledronic acid. In contrast, in the subgroup analysis of the study by Henry et al. [17], in which the zoledronic acid dosing regimen was appropriately adjusted for renal function and daily supplementation with calcium and vitamin D was strongly recommended, the incidence rate of hypocalcemia was 3.5% among patients receiving zoledronic acid and 5.7% among those receiving denosumab. Renal dysfunction was observed in 5.6% of the patients receiving zoledronic acid in the study by Lipton et al. [6] and in 7.1% of patients receiving denosumab and 2.4% receiving zoledronic acid in the subgroup analysis of Henry et al. [17]. Data on hypocalcemia and renal dysfunction were not available for the study by Broom et al. [15].

### OS

All three of the abovementioned studies reported OS, but the absolute numbers of events were not available from the studies by Lipton et al. [6] and Broom et al. [15]. Both studies showed improved OS with zoledronic acid (9.8 months for zoledronic acid versus 7.2 months for placebo; 13.6 months for zoledronic acid plus everolimus versus 10.7 months for everolimus alone), but the difference was not significant (*P* = 0.179 and 0.088, respectively). In the subgroup analysis by Henry et al. [17], 28 of 70 patients (40%) and 24 of 85 patients (28%) died of various causes in the denosumab and zoledronic acid arms, respectively. The median OS for the denosumab and zoledronic acid arms was 23.4 months versus “not reached” (HR 1.44; 95% CI: 0.84–2.51), and there was no significant difference between the two groups.

### PFS

No studies reported PFS. We calculated time-to-progression (TTP) using IPD of the RCC subgroup from the study by Henry et al. [17], which showed that 47 of 70 patients (67%) and 53 of 85 patients (62%) developed progression or died in the denosumab and zoledronic acid arms, respectively; furthermore, the median TTP for denosumab and zoledronic acid was 7.5 months versus 11.2 months (HR: 1.26; 95% CI: 0.85–1.87), respectively, but the difference was not statistically significant.

### Health-related QOL

Broom et al. [15] found better severity and interference scores in the Brief Pain Inventory (BPI) in patients receiving zoledronic acid plus everolimus compared to those receiving everolimus alone, although the difference did not reach statistical significance (*P* = 0.054 and 0.055, respectively), whereas no detectable effect was seen in the Functional Assessment of Cancer Therapy–Bone Pain scores (*P* = 0.53). In the subgroup analysis of the study by Henry et al. [17], patients receiving denosumab had significantly improved BPI severity and Functional Assessment of Cancer Therapy–General scores compared to those receiving zoledronic acid (*P* = 0.045 and 0.016, respectively), although there were no significant differences in BPI interference and Euro QOL 5 Dimension scores (*P* = 0.5 and 0.47, respectively).

### Cost effectiveness

None of the studies reported the cost effectiveness.

## DISCUSSION

A total of three RCTs (overall, 259 patients), in which zoledronic acid or denosumab was administered as BMA treatment, were included in this review. The pooled results of two RCTs that compared zoledronic acid with a placebo or no zoledronic acid suggest that zoledronic acid reduces the risk of SREs by 68% in patients with RCC with bone metastases, and there was no heterogeneity across studies. With regard to denosumab, we identified only one RCT comparing denosumab and zoledronic acid; the subgroup analysis of 155 patients with RCC in this study showed a favorable efficacy of denosumab in terms of SREs, but the difference between the two groups did not meet the conventional level of statistical significance.

To date, the role of BMAs in the treatment of bone metastases in patients with RCC remains controversial. Nevertheless, no systematic review has thus far evaluated the efficacy and safety of BMAs in this setting. As mentioned above, although an early subgroup analysis of a phase III trial suggested a superiority of zoledronic acid over placebo in terms of SREs [6], a recent post-hoc analysis of a larger number of patients who were treated in 8 phase II and phase III trials of various targeted agents demonstrated that bisphosphonate therapy did not impact survival or SRE prevention and was associated with increased toxicity [3]. However, this study was retrospective in design, all the RCTs included were designed to evaluate the efficacy of targeted agents (not BMAs), “patients with bone-only metastases were excluded,” and 72% of participants did not have bone metastases. Moreover, it “was not designed to capture SREs” and the rate of SREs was much lower than the previously reported rates [6]. With respect to denosumab, our review is the first to analyze the IPD of the RCC subgroup from the study by Henry et al. [17]. Considering these findings, we believe that our review highlights the best currently available evidence on the efficacy of BMAs for the treatment of bone metastases in patients with RCC.

In the current EAU guidelines, no recommendation is made for the use of BMAs in patients with RCC with bone metastases [7]. However, our moderate-quality evidence indicates that zoledronic acid significantly reduces the SRE risk among patients with bone metastases of RCC. Furthermore, zoledronic acid was generally well tolerated even when used in combination with everolimus, although “a relatively high number of zoledronic acid dose omissions, reductions, and cessations were observed, owing to drops in creatinine clearance and hypocalcemia” [15]. Based on these findings, zoledronic acid, with appropriate supplementation of calcium and vitamin D and adjustments of dosing regimen for renal function, is a practical option for the treatment of bone metastases of RCC, even in the targeted-therapy era. The current guidelines for the treatment of bone metastasis of various malignancies also recommend a daily supply of vitamin D (natural form; 400 IU) and oral calcium (500 mg) with careful monitoring of serum calcium level, especially in denosumab-treated patients, as well as dose modification based on creatinine clearance rates with careful monitoring of serum creatinine level, especially in zoledronic acid-treated patients [4, 18]. It should be noted that safety in patients with severe renal impairment is unknown, as these individuals were excluded from the studies.

The role of denosumab in patients with RCC with bone metastases needs to be explored further. As mentioned above, to our knowledge, only one RCT evaluated the efficacy of denosumab in patients with advanced cancer and myeloma including RCC [16, 17]. The subgroup analysis of 155 patients with RCC showed no significant differences in SREs between denosumab and zoledronic acid groups, whereas the overall study of 1,776 patients and an ad-hoc analysis of a subgroup of 1,597 patients with solid tumors demonstrated favorable efficacy of denosumab in SREs compared with zoledronic acid [16, 17]. Robust and adequately powered RCTs with adequate duration of follow-up are required to detect potential differences between denosumab and zoledronic acid in patients with RCC. A meta-analysis of individual data may be particularly useful for demonstrating the mechanisms by which the type of bone metastases (i.e., osteolytic, osteoblastic, or mixed) and subtype of RCC (i.e., clear cell RCC or non-clear cell RCC) modify the effect of BMAs. Future studies should examine the cost effectiveness of treatment while considering patients’ values and preferences. In addition, evaluation of the efficacy and safety of BMAs in combination with sunitinib, pazopanib, or nivolumab, which are recommended as first- and second-line therapy for advanced RCC [7], will be clinically useful.

The strengths of this review include its rigorous methodology that followed a written, a priori protocol developed according to the PRISMA statement [13], including a comprehensive search of evidence; duplicate assessment of eligibility, RoB, and data abstraction; and use of the GRADE approach for assessing the certainty of evidence. In addition, we only included RCTs, all of which were multicenter studies and most of which involved multiethnic patients.

Despite our important findings, this study has a few limitations that need to be addressed. First, there was clinical and methodological (but not statistical) heterogeneity across studies in terms of the study year and treatment outside of the study protocol. Second, although the search was thorough, it is possible that there are unpublished studies that have not been identified, but the small number of studies identified precludes the detection of any publication bias. Although we retrieved as much data as possible by requesting pharmaceutical companies and study authors to supply all available information, we cannot be completely sure that some data are not missing, especially in studies with negative findings. Finally, the study by Henry et al. [17] was stratified by factors related to SRE risk, but not controlled for factors related to disease outcomes; due to this, the subgroup analysis with a small sample size could have led to significant baseline imbalance, including confounding and prognostic factors, in the two groups. In our post-hoc subgroup analysis, clinically important differences in survival outcomes were noted between denosumab and zoledronic acid, even without statistically significant differences, but these outcomes were similar in the two groups overall [17].

## MATERIALS AND METHODS

### Search strategy, selection of studies, and data extraction

The protocol for this review has been registered (www.crd.york.ac.uk/PROSPERO; registration number CRD42016032742), and the search strategy is outlined in the Supplementary document. In brief, databases including MEDLINE and the Cochrane Central Register of Controlled Trials were systematically searched up to January 2017 without language restriction. To identify completed and ongoing studies, we systematically searched both the WHO International Clinical Trials Registry Platform and ClinicalTrials.gov. The search was complemented by additional sources including the reference lists of included studies. Two reviewers (KO and MH) screened all abstracts and full-text articles independently. Disagreement was resolved by discussion, and where no agreement was reached, a third independent reviewer (TAF) acted as an arbiter.

### Types of study design included

RCTs were eligible, including randomized phase II and phase III trials.

### Types of participants included

The study population consisted of patients aged ≥ 18 years who were diagnosed with RCC and bone metastases. Patients who received or did not receive other systemic and/or local therapies were included.

### Types of interventions included

The agents considered included pamidronate, zoledronic acid, denosumab, and other BMAs identified during the search. Valid eligible comparators included placebo, another drug (from the abovementioned drugs), or the same drug of different dose.

### Types of outcome measures included

The primary outcomes were the proportion of patients with one or more SREs and the proportion of those with serious adverse events (AEs) classified as grade 3, 4, or 5 according to the National Cancer Institute's Common Terminology Criteria for Adverse Events (CTCAE), Version 4.0 [10]. We accepted any definitions that were considered similar to this version. Secondary outcomes included time to first SRE; skeletal morbidity rate (SMR), defined as the ratio of the number of SREs for each subject divided by the subject's time at risk in years; overall AEs; OS; PFS; health-related QOL; and cost effectiveness.

### Data extraction

A data-extraction form was developed specifically to collect information on the methods, participants, interventions and other treatments, primary and secondary outcomes, statistical analysis, baseline characteristics, and results. Two reviewers (KO and MH) independently extracted the data. Missing, unclear, or important additional data were requested from primary study authors.

### Assessment of risk of bias and certainty of evidence

The standard Cochrane Collaboration risk of bias tool [11] was used to assess risk of bias (RoB) in the selected studies. The Grading of Recommendation Assessment, Development, and Evaluation (GRADE) approach was used to assess the certainty of the evidence for each pooled analysis. Two reviewers (KO and MH) independently assessed the evidence, and any disagreement was resolved by discussion or consultation of a third assessor (YT).

### Data analysis

Descriptive statistics were used to summarize baseline characteristics. A quantitative synthesis (i.e., meta-analysis) was performed if methodologically appropriate. The random-effects model was used due to the anticipated clinical heterogeneity of participants and interventions. For time-to-event data, we used “O – E” (observed minus expected) and “V” (variance) statistics or hazard ratios (HR) with 95% confidence intervals (CIs) for each study. If these values were not reported for a given study, we calculated them from available statistics, if possible, using the methods described by Tierney et al. [12]. Statistical heterogeneity between studies was assessed by visual inspection of plots of the data, the chi-square test for heterogeneity, and the *I*^2^ statistic. Analysis was performed using Cochrane Review Manager (RevMan) 5.3 (Cochrane Tech, London, UK). When meta-analysis was not feasible, a narrative synthesis was provided instead, incorporating data on HR with 95% CIs and median OS or PFS for time-to-event data and proportions (%) for categorical data. The mean and standard deviation were used to summarize continuous outcome data and were compared using the mean difference and 95% CI.

## CONCLUSIONS

SREs are among the most-common and relevant complications in patients with advanced RCC with bone metastases, and developing an optimal strategy for preventing SREs is crucial. In this systematic review and meta-analysis, we established moderate-quality evidence to prove that treatment with zoledronic acid significantly reduces the risk of SREs for patients with RCC with bone metastases. Further studies should determine the efficacy and safety of denosumab.

## SUPPLEMENTARY MATERIALS FIGURES AND TABLES









## Abbreviations

CI: confidence interval
GRADE: Grading of Recommendation Assessment, Development, and Evaluation
HR: hazards ratio
OS: overall survival
PFS: progression-free survival
RCC: renal cell carcinoma
RCT: randomized controlled trial
