# Supplementary material for: Comparative efficacy and safety of bone-modifying agents for the treatment of bone metastases in patients with advanced renal cell carcinoma: a systematic review and meta-analysis
Source: Oncotarget. 2017 Aug 18;8(40):68890–8. doi: 10.18632/oncotarget.20323 (PMC5620305; doi:10.18632/oncotarget.20323)
Supplement: Supplementary file 1 [file oncotarget-08-68890-s001.pdf]

Comparative efficacy and safety of bone-modifying agents for the treatment of bone metastases in patients with advanced renal cell carcinoma: a systematic review and meta-analysis

SUPPLEMENTARY MATERIALS

BACKGROUND

Description of the condition

Bone is known to be the most-common site of metastatic solid-cancer spread. Approximately 30% of patients with metastatic renal cell carcinoma (mRCC) develop bone metastases. Bone metastases cause significant morbidity due to pain, pathological fracture, hypercalcemia, and spinal cord compression as well as contribute to mortality.

The pathophysiology of bone metastases includes increased bone turnover and imbalance in and uncoupling of resorption and remodeling [1]. Osteoclasts are primarily responsible for bone resorption of lytic metastases [2] and are involved in a complex osteolytic cycle that involves parathyroid hormone-related peptide (PTHrP), receptor activator of nuclear factor (NF)-κB ligand (RANK-L), osteoprotegrin (OPG), transforming growth factor-beta (TGF-b), and many other transcription factors. Tumors secrete PTHrP that stimulates osteoblasts, which respond by secreting RANK-L and inhibiting production of OPG. The increased RANK-L/OPG gradient activates osteoclasts, which in turn produce TGF-b and other growth factors, all of which have a profound effect on tumor growth. Thus, tumors and osteoclasts are engaged in a self-perpetuating cycle, wherein the tumor and osteoclasts fuel each other [3].

Description of the intervention

Before the era of bisphosphonates, the management of symptomatic bone disease depended on analgesics, radiotherapy, endocrine therapy, and chemotherapy. Despite these effective treatments, progressive skeletal destruction often leads to continuing symptoms and deterioration of the quality of life (QOL) [4]. Bisphosphonates inhibit osteoclastic bone resorption [5] and are effective in conditions characterized by osteoclast-mediated bone resorption such as Paget's disease and osteoporosis [6]. In malignancies, they are the standard treatment for tumor-induced hypercalcemia [7].

How the intervention might work

Randomized controlled trials (RCTs) have shown that in multiple myeloma, breast cancer, and prostate cancer, bisphosphonates reduce bone pain, improve QOL, and decrease the number of and time to skeletal-related events (SREs) [8, 7]. In addition, pre-clinical research has suggested that bisphosphonates exhibit anti-tumor activity by inhibiting cell migration and invasion and inducing apoptosis in breast cancer cells [9].

Why it is important to do this review

Examination of the adjuvant role of bisphosphonates in patients with mRCC is important. In addition to bisphosphonates, many novel agents that specifically target the vicious cycle of bone metastases are being developed. Although many agents are still in the early stages of drug development, the RANK-L inhibitor denosumab has already completed phase III clinical trials in breast and prostate cancers [10, 11]. With superior efficacy and good tolerability, denosumab is expected to have important applications in clinical practice for patients with mRCC.

An early subgroup analysis of 46 patients with RCC enrolled in a phase III trial suggested a reduction in the SREs and a trend toward improved overall survival (OS) with zoledronic acid compared to placebo. However, this finding has been questioned by a recent post-hoc analysis of pooled data of 2,749 patients with bone metastases from 8 phase II or phase III trials, in which bisphosphonate treatment was not associated with improved progression-free survival (PFS) or OS. In the current European Association of Urology guidelines on RCC, there is no recommendation for the use of bisphosphonates and other bone-modifying agents in patients with bone metastases.

The aim of this systematic review was to identify, describe, and summarize high-quality evidence regarding the use of bisphosphonates and denosumab in patients with mRCC.

OBJECTIVES

To determine the comparative efficacy and safety of all available bisphosphonates and denosumab for the treatment of bone metastases in patients with mRCC.

MATERIALS AND METHODS

Criteria for studies

Types of studies

We will include all relevant RCTs that examined the use of bisphosphonates and/or other bone-modifying agents in the treatment of bone metastases in patients with RCC. Although we will include cross-over studies, we will use only the first phase to avoid carry-over effects. In addition, we will include cluster-randomized studies if the intracluster correlation coefficient is reported and exclude quasi-randomized studies.

Types of participants

We will include adults aged ≥18 years in whom RCC with bone metastases is histologically verified. Patients of any gender and ethnicity who are treated in any setting will be included. Patients may or may not have received other systemic and/or local therapies.

Types of interventions

A bisphosphonate or denosumab must be part of the therapeutic regimen of at least one study arm. Control conditions will include placebo, another drug (from the abovementioned drugs), or the same drug of different dose.

Types of outcome measures

Primary outcomes

1. Proportion of SREs measured as the proportion of patients with one or more SREs, which are defined as pathologic fracture, spinal cord compression, hypercalcemia, radiation therapy, or bone surgery.
2. Serious adverse events (AEs) classified as grade 3, 4, or 5 according to the National Cancer Institute's Common Terminology Criteria for Adverse Events (CTCAE), Version 4.0 [12]. We will accept any definitions that are similar to this version.

Secondary outcomes

1. Time to the first SRE measured as the duration from the beginning of treatment to the time of the first SRE.
2. Skeletal morbidity rate (SMR) defined as the ratio of the number of SREs for each subject divided by the subject's time at risk in years. For example, if a study follows 1000 patients for 1 year, and among those 1000 patients, 350 SREs occur, then the SMR value would be 0.35 SREs/year. If multiple events occur within 1 year, these values are included within the ratio.
3. AEs classified as grade 1 or 2 according to the CTCAE, Version 4.0. We will accept any definitions that are similar to this version.
4. OS defined as the time from enrollment in the study to death.
5. PFS defined as the time from enrollment in the study to progression or death. Progression is assessed on the basis of the response-evaluation criteria in solid tumors (RECIST) [13], version 1.0, guidelines by use of computed tomography.
6. Health-related QOL evaluated by change in a validated QOL-measuring scoring platform such as the 36-Item Short Form Health Survey (SF-36) [14], Euro QOL 5 Dimension (EQ-5D).
7. Cost effectiveness measured as the incremental cost effective ratio (ICER).

Search methods for identification of studies

Electronic searches

We will conduct a literature search to identify all published and unpublished randomized controlled trials. The literature search will identify potential studies in all languages. We will translate the non-English language papers and fully assess them for potential inclusion in the review, as necessary.

We will search the MEDLINE and Cochrane Central Register of Controlled Trials (CENTRAL) electronic databases from inception to present to identify potential studies (Appendix 1, 2). For ongoing studies, in addition to the WHO International Clinical Trials Registry Platform (ICTRP), we will systematically search the Clinical Trials.gov database (Appendix 3, 4).

Other sources

We will also search registers of pharmaceutical industry trials and bibliographies for any additional trials that may have been missed in the initial database search. Furthermore, we will manually search abstracts in the proceedings of the periodic major meetings and reference lists of all eligible trials identified.

Data collection and analysis

Selection of studies

Two review authors (KO and MH) will independently examine the titles and abstracts of all the potential studies identified as a result of the electronic search and determine whether the study is relevant. Thereafter, we will retrieve full texts of all the relevant studies, and the abovementioned two review authors will independently assess them for inclusion. If the study is to be excluded, the review authors will record the reasons for exclusion. We will resolve any disagreement through discussion among review authors or, if required, consultation with a third person (TF). We will identify and exclude duplicates of the same study, so that each study, rather than each report, is a unit of interest in the review. Agreement between the two review authors (KO and MH) in determining study eligibility will be reported as percentage agreement and Cohen's weighted kappa.

When it is impossible to assess the eligibility of the study due to lack of information, the study will be categorized as "study awaiting assessment" until further information can be obtained. We will record the selection process in sufficient detail to complete a Preferred Reporting Items for Systematic Reviews and Meta-Analyses (PRISMA) flow diagram and table for the characteristics of excluded studies.

Data extraction and management

At least two review authors (KO and MH) will use a structured, pilot-tested, data-extraction form (Appendix 6) to independently collect data from the included studies. The data collected will include methods, participants, interventions, other treatments, primary and secondary outcomes, statistical analysis, baseline characteristics, and results. Agreement between the data extractors will be reported as percentage agreement and weighted kappa. Any disagreement will be resolved thorough discussion or, if required, consultation with a third person (TF).

Assessment of risk of bias in included studies

Two review authors (KO and MH) will independently assess the risk of bias for each study using the criterion outlined in the *Cochrane Handbook for Systematic Reviews of Interventions* [15]. We will assess the risk according to the following domains (Appendix 7 presents the criteria for determining risk of bias):

1. Random sequence generation
2. Allocation concealment
3. Blinding of participants and personnel
4. Blinding of outcome assessment
5. Incomplete outcome data
6. Selective outcome reporting
7. Other bias (including blinding of data analysis, pre-randomization administration of an intervention, inappropriate administration of an intervention or co-intervention, baseline imbalance, early end, supplement for the dropouts by providing additional recruits, recruit of additional participants from a subgroup showing more or less benefit, deviation from the study protocol in a way that does not reflect clinical practice, differential diagnostic activity, and pharmaceutical funding and/or support)

The risk of bias in each domain and overall will be assessed and categorized into the following:

- Low risk of bias: plausible bias unlikely to seriously alter the results
- Unclear risk of bias: plausible bias that raises some doubt about the results
- High risk of bias: plausible bias that seriously weakens confidence in the results

Agreement between the two review authors with regard to the risk of bias will be reported as percentage agreement and weighted kappa. Any disagreement will be resolved by discussion or consultation with a third assessor (YT). We will provide a quote from the study report together with a justification for our judgment in the risk of bias table. We will summarize the risk of bias judgment across different studies for each of the domains listed. When information on risk of bias is related to unpublished data or correspondence with a trialist, we will note this in the risk of bias table.

Measures of treatment effect

1. Dichotomous outcomes

We will analyze dichotomous data as odds ratio (OR) with 95% confidence interval (CI) because of its favorable mathematical properties. However, because ORs can be difficult to interpret, these pooled ORs will be converted to risk ratios (RRs) using the following formula provided in the Cochrane Handbook for Systematic Reviews of Interventions [15] and will be presented in the "Summary of findings" tables for ease of interpretation.

RR = OR/(1 - ACR) × (1 - OR), where ACR is the assumed control risk.

2. Continuous outcomes

When studies use the same outcome measure for comparison, data will be pooled by calculating the mean difference (MD) with 95% CI. When different measures are used to assess the same outcome, data will be pooled with standardized MD with 95% CI.

3. Time-to-event data

We will use hazard ratios (HRs) to estimate the size of intervention differences, where available. For our meta-analysis of time-to-event outcomes in Review Manager 5.3 (RevMan) [16], we will use "O - E" (observed minus expected) and "V" (variance) statistics or HRs for each trial. If those values are not reported for a given trial, we will calculate them from available statics, if possible, using the methods described in a previous study [17].

4. Change versus endpoint data

We will use change data only when endpoint data are unavailable.

5. Skewed data

(a) Endpoint data

When a scale starts from the finite number zero, we will subtract the lowest-possible value from the mean and divide this value by the standard deviation (SD). If this value is <1, it strongly suggests a skew, and the study will be excluded from meta-analytic pooling and be narratively presented. If this ratio is >1 but <2, there is a possibility of a skew. In this case, we will include the study and test whether its inclusion or exclusion substantially changed the results. If the ratio is >2, the study will be included because a skew is less likely [18, 15].

(b) When continuous data are presented on a scale that includes the possibility of a negative value (such as change data), it is difficult to determine whether data are skewed. We plan to include such studies because change data tend to be less skewed and excluding these studies would lead to bias because not all the available information would be used.

(c) A common way that trialists indicate when they have skewed data is by reporting medians and interquartile ranges. When we encounter this situation, we will note that the data are skewed, and the study will be excluded from meta-analytic pooling and be narratively summarized.

Unit of analysis issues

1. Cluster-randomized trials

Cluster-randomized trials will only be included as long as proper adjustment for the intra-cluster correlation (ICC) is conducted in accordance with chapter 16.3.5 of the Cochrane Handbook for Systematic Reviews of Interventions [15]. For dichotomous data, we will apply the design effect and calculate effective sample size and number of events using ICC and the average cluster size. If ICC has not been reported, we will use ICC of similar studies as a substitute. For continuous data, only the sample size will be reduced; means and SD will remain unchanged.

2. Cross-over trials

Trials employing a cross-over design will be included in the review, but only data from the first active treatment phase will be used to avoid carry-over effects.

3. Studies with multiple treatment groups

Where multiple trial arms are reported in a single trial, we will include only the relevant arms. For dichotomous outcomes, data from different dosages of the same relevant active intervention arms will be collapsed into a single arm for comparison, or when a study involves two different relevant active intervention arms and a placebo arm, data from the placebo arm will be split equally between comparator arms. For continuous outcomes, means and SDs will be combined using methods described in the Cochrane Handbook for Systematic Reviews of Interventions [15] as follows:

	**Group 1 (e.g., men)**	**Group 2 (e.g., women)**	**Combined groups**
**Sample size**	N_1	N_2	$N_1 + N_2$
**Mean**	M_1	M_2	$\frac{N_1M_1 + N_2M_2}{N_1 + N_2}$
**SD**	SD_1	SD_2	$\sqrt{\frac{(N_1 - 1)SD_1^2 + (N_2 - 1)SD_2^2 + \frac{N_1N_2}{N_1 + N_2}(M_1^2 + M_2^2 - 2M_1M_2)}{N_1 + N_2 - 1}}$

SD, standard deviation.

Dealing with missing data

1. Missing participants

(a) Dichotomous data

All data will be analyzed, as far as possible, on the basis of the intention-to-treat principle: dropouts will be always included in this analysis. In cases where participants withdrew from the trial before the endpoint and the original authors did not impute it appropriately, we will impute missing data assuming a poor outcome (worst-case scenario) for missing individuals. Any assumptions and imputations to handle missing data will be clearly described, and the effect of imputations will be explored by sensitivity analyses.

(b) Continuous data

If data can be assumed to be missing at random, we will analyze only the available data. To assess how sensitive the results are to the assumptions made, we will perform sensitivity analysis including only studies in which we can assume that there were no missing data or that the missing data occurred at random.

2. Missing data

We will contact investigators or study sponsors to obtain missing numerical outcome data where possible (e.g., when a study is identified as abstract-only).

3. Missing statistics

When only the standard error (SE) or *P* value is reported, SDs will be calculated according to a previous study [17]. In the absence of supplemental data after requests to the authors, the SDs will be calculated from CIs, *t*-values, or *P* values as mentioned in the equation below and described in Chapter 7.7.3.2 of the *Cochrane Handbook for Systematic Reviews of Interventions* [15] or they will be imputed from other studies in the meta-analysis according to a previously validated method [19]. We will examine the validity of these imputations in a sensitivity analysis.

$$SD = SE \times \sqrt{N}, \quad SD = \sqrt{N} \times \frac{UL - LL}{2 \times (t - value)}$$

where N denotes the sample size of each group, UL denotes the upper limit of the confidence interval, and LL denotes the lower limit of the confidence interval. The *t*-value for the 95% confidence interval will be calculated in Microsoft Excel using the formula $tinv(1-0.95, N-1)$. In cases where the actual *P* values obtained from *t*-tests are reported, the corresponding *t*-value will be obtained from a table of the *t*-distribution.

Assessment of heterogeneity

We will conduct visual inspection of the forest plot to investigate the presence and nature of statistical heterogeneity. In addition, we will assess the presence of statistical heterogeneity using the Chi-square test and I^2 statistic. Since the Chi-square test has low power when studies have small sample size or are few in number, we will use a *P* value of 0.10 conservatively to determine statistical significance. I^2 values will be roughly interpreted according to the guide in Chapter 9.5.2 of the *Cochrane Handbook for Systematic Reviews of Interventions* [15]. However, the importance of the observed I^2 depends on the magnitude and direction of treatment effects and the strength of evidence for heterogeneity. To provide an indication of the spread of true investigation effects, we will report between-study variance in a random-effects meta-analysis using Tau-squared values.

Assessment of reporting bias

The impact of reporting biases will be minimized by undertaking comprehensive searches of multiple sources (including trial registries), increasing efforts to identify unpublished materials, and including non-English language publications. We will try to identify outcome-reporting bias in trials by recording all trial outcomes, planned and reported, and noting where outcomes were missing. When evidence of missing outcomes is found, we will attempt to obtain any available data directly from the authors. We will first assess the heterogeneity by visual inspection of the forest plot. If the number of eligible studies is ≥ 10, Egger's test will be used to investigate the potential influence of reporting biases and small-study effects.

Data synthesis

Given the likelihood of the heterogeneity between studies, data will be pooled using a random-effects model.

Subgroup analysis and investigation of heterogeneity

We will perform subgroup analyses to explore whether the effect of the intervention differs according to the following variables for the primary outcomes only (proportion of SRE and severe adverse events):

1. Type of bone-modifying agents: zoledronate, denosumab, pamidronate, others
2. Study populations: elderly (≥ 65 years) and non-elderly (< 65 years), those with and without renal insufficiency
3. Year of publication (measured as a continuous variable) as a general proxy for various aspects (e.g., trial quality and quality of medical care)

Sensitivity analysis

We plan to perform the following sensitivity analyses for the primary outcomes only (proportion of SRE and severe AEs) to assess the robustness of our conclusions:

1. Restrict the inclusion in the analyses only to studies considered to be at low risk of selection and detection bias (i.e., adequate allocation-sequence generation, adequate allocation concealment, and blinding of assessor)
2. Exclude studies whose missing data were imputed
3. Use a fixed-effect model instead of a random-effects model

Summary of findings table

We will create a summary of findings table using the following outcomes: Proportion of SRE, AEs, time to first SRE, SMR, OS, PFS, and QOL. We will use Grading of Recommendations Assessment, Development and Evaluation (GRADE) considerations (study limitations, consistency of effect, imprecision, indirectness, and publication bias) to assess the certainty of a body of evidence, as it relates to the studies that contribute data to the meta-analysis for the pre-specified outcomes. We will use methods and recommendations described in Section 8.5 and Chapter 12 of the *Cochrane Handbook for Systematic Reviews of Interventions* [15]. We will determine whether there is any additional outcome information that was not incorporated into the meta-analysis, note this in the comments, and state if it supports or contradicts the information obtained from the meta-analysis.

REFERENCES

1. Kanis JA. Bone and cancer: pathophysiology and treatment of metastases. Bone. 1995; 17:101S–05S.
2. Mundy GR. Mechanisms of bone metastasis. Cancer. 1997; 80:S1546–56.
3. Kozlow W, Guise TA. Breast cancer metastasis to bone: mechanisms of osteolysis and implications for therapy. J Mammary Gland Biol Neoplasia. 2005; 10:169–80.
4. Mundy GR. Mechanisms of osteolytic bone destruction. Bone. 1991; 12:S1–6.
5. Rogers MJ, Watts DJ, Russell RG. Overview of bisphosphonates. Cancer. 1997; 80:S1652–60.
6. Russell RG, Rogers MJ. Bisphosphonates: from the laboratory to the clinic and back again. Bone. 1999; 25:97–106.
7. Body JJ, Bartl R, Burckhardt P, Delmas PD, Diel IJ, Fleisch H, Kanis JA, Kyle RA, Mundy GR, Paterson AH, Rubens RD, and International Bone and Cancer Study Group. Current use of bisphosphonates in oncology. J Clin Oncol. 1998; 16:3890–99.
8. Bloomfield DJ. Should bisphosphonates be part of the standard therapy of patients with multiple myeloma or bone metastases from other cancers? An evidence-based review. J Clin Oncol. 1998; 16:1218–25.
9. Hiraga T, Williams PJ, Ueda A, Tamura D, Yoneda T. Zoledronic acid inhibits visceral metastases in the 4T1/luc mouse breast cancer model. Clin Cancer Res. 2004; 10:4559–67.
10. Fizazi K, Carducci M, Smith M, Damião R, Brown J, Karsh L, Milecki P, Shore N, Rader M, Wang H, Jiang Q, Tadros S, Dansey R, Goessl C. Denosumab versus zoledronic acid for treatment of bone metastases in men with castration-resistant prostate cancer: a randomised, double-blind study. Lancet. 2011; 377:813–22.
11. Stopeck AT, Lipton A, Body JJ, Steger GG, Tonkin K, de Boer RH, Lichinitser M, Fujiwara Y, Yardley DA, Viniegra M, Fan M, Jiang Q, Dansey R, et al. Denosumab compared with zoledronic acid for the treatment of bone metastases in patients with advanced breast cancer: a randomized, double-blind study. J Clin Oncol. 2010; 28:5132–39.
12. National Cancer Institute. Cancer Therapy Evaluation Program. NIH. http://ctep.cancer.gov/protocolDevelopment/electronic_applications/ctc.htm#ctc_40. Published March 2003. Updated November 14, 2016. Accessed March 4, 2017.
13. Eisenhauer EA, Therasse P, Bogaerts J, Schwartz LH, Sargent D, Ford R, Dancey J, Arbuck S, Gwyther S, Mooney M, Rubinstein L, Shankar L, Dodd L, et al. New response evaluation criteria in solid tumours: revised RECIST guideline (version 1.1). Eur J Cancer. 2009; 45:228–47.
14. Ware JE Jr, Sherbourne CD. The MOS 36-item short-form health survey (SF-36). I. Conceptual framework and item selection. Med Care. 1992; 30:473–83.
15. The Cochrane Collaboration. Cochrane Handbook for Systematic Reviews of Interventions Version 5.1.0. Updated March 2011. Available from http://handbook.cochrane.org/
16. Manager R. (RevMan) [Computer program]. Version 5.1. Copenhagen: The Nordic Cochrane Centre, The Cochrane Collaboration; 2011.
17. Tierney JF, Stewart LA, Ghersi D, Burdett S, Sydes MR. Practical methods for incorporating summary time-to-event data into meta-analysis. Trials. 2007; 8:16.
18. Altman DG, Bland JM. Detecting skewness from summary information. BMJ. 1996; 313:1200.
19. Furukawa TA, Barbui C, Cipriani A, Brambilla P, Watanabe N. Imputing missing standard deviations in meta-analyses can provide accurate results. J Clin Epidemiol. 2006; 59:7–10.

Appendix 1: MEDLINE via PubMed search strategy

#1 randomized controlled trial [pt] OR controlled clinical trial [pt] OR randomized [tiab] OR placebo [tiab] OR drug therapy [sh] OR randomly [tiab] OR trial [tiab] OR groups [tiab] NOT (animals [mh] NOT humans [mh]) 3415465 (01/27/2017)
#2 kidney neoplasms [mh] OR (kidney AND neoplasms) OR “kidney neoplasms” OR (kidney AND cancer) OR “kidney cancer” OR carcinoma, renal cell [mh] OR (carcinoma AND renal AND cell) OR “renal cell carcinoma” OR (renal AND cell AND carcinoma) OR (kidney AND tumor) OR “kidney tumor” 141420 (01/27/2017)
#3 diphosphonates [mh] OR diphosphonates OR bisphosphonate OR pamidronate OR “zoledronic acid” OR zoledronate OR denosumab 27411 (01/27/2017)
#4 #1 AND #2 AND #3 293 (01/27/2017)

Appendix 2: CENTRAL search strategy

#1 MeSH descriptor [Kidney Neoplasms] explode all trees
#2 Kidney near cancer*
#3 Kidney near neoplasm*
#4 Kidney near tumour*
#5 Kidney near tumor*
#6 Kidney near malignan*
#7 Renal near cancer*
#8 Renal near neoplasm*
#9 Renal near tumour*
#10 Renal near tumor*
#11 Renal near malignan*
#12 #1 or #2 or #3 or #4 or #5 or #6 or #7 or #8 or #9 or #10 or #11 2413
#13 MeSH descriptor [Carcinoma, Renal Cell] explode all trees
#14 renal near cell near carcinom*
#15 #13 or #14 1232
#16 #12 or #15 2801
#17 MeSH descriptor [Diphosphonates] explode all trees
#18 Diphosphonate
#19 Diphosphanate
#20 Bisphosphonate
#21 Bisphosphanate
#22 Pamidronate
#23 Zoledronate
#24 Zoledronic near acid
#25 Denosumab
#26 #17 or #18 or #19 or #20 or #21 or #22 or #23 or #24 or #25 3433
#27 #16 and #26 49 (Trials 32) (01/27/2017)

Appendix 3: ClinicalTrials.gov search strategy

#1 (kidney OR renal) AND (cancer OR neoplasm OR carcinoma) AND (diphosphonates OR bisphosphonates OR pamidronate OR zoledronate OR zoledronic acid OR denosumab) 31 (01/27/2017)

Appendix 4: WHO ICTRP search strategy (01/27/2017)

1. Renal cell carcinoma AND bisphosphonate*	1
2. Renal cell carcinoma AND bisphosphonate	0
3. Renal cell carcinoma AND bisphosphonates	1
4. Renal cell carcinoma AND biphosph*	0
5. Renal cell carcinoma AND diphosphonate*	0
6. Renal cell carcinoma AND diphosphonate	1
7. Renal cell carcinoma AND diphosphonates	1
8. Renal cell carcinoma AND diphosph*	0
9. Kidney cancer AND bisphosphonate*	2
10. Kidney cancer AND bisphosphonate	0

Summary of search results

No. of records identified through MEDLINE via PubMed: 293 (01/27/2017)
No. of records identified through CENTRAL: 49 (01/27/2017)
No. of records identified through ClinicalTrials.gov: 32 (01/27/2017)
No. of records identified through WHO ICTRP: 2 (01/27/2017)

Appendix 5: Cohen's weighted kappa statistics in 10 selected articles

		Reviewer 2			
		**Include**	**unsure**	**Exclude**	**Total**
Reviewer 1	Include	1	1	0	2
	Unsure	0	1	1	2
	Exclude	0	0	6	6
	Total	1	2	7	10

P0 (Percentage agreement) = 0.80, PE = 0.48,
Simple kappa = 0.62, weighted kappa = 0.72

Appendix 6: Data-extraction sheet. See Supplementary_ Appendix_6

Appendix 7: Criteria for judging risk of bias in the "risk of bias" assessment tool. See Supplementary_ Appendix_7

Supplementary Table 1: Summary of findings for the included studies. See Supplementary_Table 1
