# Supplementary material for: Comparative efficacy and safety of bone-modifying agents for the treatment of bone metastases in patients with advanced renal cell carcinoma: a systematic review and meta-analysis
Source: Oncotarget. 2017 Aug 18;8(40):68890–8. doi: 10.18632/oncotarget.20323 (PMC5620305; doi:10.18632/oncotarget.20323)
Supplement: Supplementary file 2 [file oncotarget-08-68890-s002.docx]

Supplementary Table 1: Summary of findings for the included studies

| Study | Comparator | Median age | Sex, male/female | Skeletal-related event (SRE) (%) | SRE HR | Time to the first SRE (months) | Skeletal morbidity rate (events/year) | OS (months) | OS HR | Time-to-progression (TTP) (months) | TTP HR | Serious adverse events (AEs) (%) | Serious AE risk ratio | Serious AE types | Brief Pain Inventory (BPI) severity | BPI interference | Functional Assessment of Cancer Therapy–Bone Pain | Functional Assessment of Cancer Therapy–General | Euro QOL 5 Dimension |
| --- | --- | --- | --- | --- | --- | --- | --- | --- | --- | --- | --- | --- | --- | --- | --- | --- | --- | --- | --- |
| BroomRJ et al. 2015  [15] | Zoledronic acid + everolimus | 69.2 | 10/5 | 60/year | 0.32 (95%CI: 0.14-0.76) | 9.6 (95%CI 4.3–15.5) | - | 13.6 (95%CI 9.6–22.2) | - | - | - | 80 | 1 (95%CI 0.24–4.18) | Osteonecrosis: 0/15 | -1.10 (95%CI -2.22–0.02) | -1.25 (95%CI -2.53–0.03) | 3.61 (95%CI -8.10–15.32) | - | - |
|  | Everolimus | 70.6 | 14/1 | 93/year |  | 5.2 (95%CI 1.6–8.2) | - | 10.7 (95%CI 3.5–14.7) |  | - |  | 80 |  | Osteonecrosis: 0/15 |  |  |  |  |  |
|  |  |  |  |  |  |  |  |  |  |  |  |  |  |  |  |  |  |  |  |
| Henry D et al. 2014  [16] | Denosumab | 59 | 57/13 | 35.6/year | 0.70 (95%CI 0.43-1.14) | NR | - | 23.4 (95%CI 17.6–NR) | 1.44 (95%CI 0.84–2.51) | 7.5 (95%CI 4.9–10.3) | 1.26 (95%CI 0.85–1.87) | 43 | 0.86 (95%CI 0.68–1.08) | Osteonecrosis: 0/70 Hypocalcemia : 1/70  Renal dysfunction: 2/70 | -0.29 (95%CI -0.58–-0.01) | -0.12 (95%CI -0.48–0.23) | - | 1.48 (95%CI 0.27–2.69) | 0.016 (95%CI -0.027–0.058) |
|  | Zoledronic acid | 61 | 59/26 | 53.3/year |  | 11.6 (95%CI 6.2–23.6) | - | NR |  | 11.2 (95%CI 6.9–14.2) |  | 61 |  | Osteonecrosis: 3/85 Hypocarcemia: 1/85  Renal dysfunction: 0/85 |  |  |  |  |  |
|  |  |  |  |  |  |  |  |  |  |  |  |  |  |  |  |  |  |  |  |
| Lipton A et al. 2003  [6] | Zoledronic acid | 64 | 18/9 | 37/9 months | 0.32 (95%CI 0.16-0.64) | NR | 2.68 | 9.8 | - | - | - | - | - | Osteonecrosis: 0/27 | - | - | - | - | - |
|  | Placebo | 65 | 17/2 | 74/9 months |  | 2.4 | 3.38 | 7.2 |  | - |  | - | - | Osteonecrosis: 0/19 |  |  |  |  |  |
