# Supplementary material for: Comparative efficacy and safety of bone-modifying agents for the treatment of bone metastases in patients with advanced renal cell carcinoma: a systematic review and meta-analysis
Source: Oncotarget. 2017 Aug 18;8(40):68890–8. doi: 10.18632/oncotarget.20323 (PMC5620305; doi:10.18632/oncotarget.20323)
Supplement: Supplementary file 3 [file oncotarget-08-68890-s003.docx]

**Appendix 6. Data-extraction sheet**

**DATA-EXTRACTION SHEET**

**General information**

| Study ID |  | | Report ID |  | Reviewer ID  □ KO □ MH |
| --- | --- | --- | --- | --- | --- |
| Citation |  | | | |  |
| Contact details | |  | | | |

**Eligibility**

| Study characteristics | Eligibility criteria | Eligibility criteria met? | | | Location in article (pg/fig/table) |
| --- | --- | --- | --- | --- | --- |
|  |  | Yes | No | Unclear |  |
| Types of study | Experimental study including randomized controlled trials, cluster randomized trials, or cross-over trials | □ | □ | □ |  |
| Participants and setting | Adults aged ≥18 years who have histologically confirmed renal cell carcinoma with bone metastases | □ | □ | □ |  |
| Types of intervention | A bisphosphonate or denosumab combined with any type of treatment | □ | □ | □ |  |
| Types of comparison | None of the abovementioned drugs; placebo; use of bisphosphonate or denosumab, whichever is not being used; or the same drug of different dose | □ | □ | □ |  |
| Types of outcome | Primary outcome |  |  |  |  |
|  | 1) Skeletal-related event (SRE) | □ | □ | □ |  |
|  | 2) Severe adverse events (AEs) (≥grade 3) | □ | □ | □ |  |
|  | Secondary outcome |  |  |  |  |
|  | 1) Time to the first SRE | □ | □ | □ |  |
|  | 2) Skeletal morbidity rate | □ | □ | □ |  |
|  | 3) Not severe AEs (≤grade 2) | □ | □ | □ |  |
|  | 4) Overall survival | □ | □ | □ |  |
|  | 5) Progression-free survival | □ | □ | □ |  |
|  | 6) Quality of life | □ | □ | □ |  |
|  | 7) Cost effectiveness | □ | □ | □ |  |
| This study can be □ Included □ Excluded | | | | | |
| *Reason for exclusion* |  | | | | |
| Note |  | | | | |

**Study Methods**

***1. Design*:**  Parallel　  Cross-over  Others

***2. Randomization*:**  Individual  Cluster　 Unclear  No randomization

***3. Language*:**

English  French  German  Spanish  Chinese  Others [ ]

***4. Country*:** [ ]

***5. Publication status*:**  Published　 Published as abstract　 Unpublished

***6. Allocation concealment***

Adequate: Participants and investigators enrolling participants cannot foresee assignment because one of the following, or an equivalent method, is used to conceal allocation:

1) Central allocation (including telephone, web-based, and pharmacy-controlled randomization)

2) Sequentially numbered drug containers of identical appearance

3) Sequentially numbered, opaque, sealed envelopes

Inadequate: Participants or investigators enrolling participants can possibly foresee assignments such as allocation using:

1) An open, random allocation schedule (e.g., a list of random numbers)

2) Assignment envelopes without appropriate safeguards (e.g., if envelopes are unsealed or non-opaque or not sequentially numbered)

3) Alternation or rotation

4) Any other explicitly unconcealed procedure

Unclear: Insufficient information to permit judgment of “Yes” or “No” for adequacy of concealment

***7. Blinding***

***1) Adequacy of blinding of patients***

Adequate: Use of chamber rooms of either intervention or control treatment and integrity of blinding is tested; for example, asking patients to guess their allocated group confirmed that the patients could not guess beyond chance

Adequate: Use of chamber rooms or state “double blind” or declare the blinding of patients, but no test of blinding

Unclear

Inadequate: Open study or statement is made about no use of identical tablets

***2) Adequacy of blinding of the treatment-prescribing physician***

Adequate: Blinding of physician is done or stated “double blind” and integrity of blinding tested

Adequate: Blinding of physician is done or stated “double blind,” but no test of blinding

Unclear

Inadequate: Not blinded; they know the allocated treatment.

***3) Adequacy of the blind outcome assessment***

Adequate: The assessor of the primary outcome defining “response” was blinded and integrity of the blinding was tested

Adequate: The primary outcome defining “response” was assessed by an independent assessor blinded to treatment allocation

Unclear

Not blind: The outcome was assessed by someone who knew the allocated treatment, including instances when the treating physician assessed the primary outcome

***8. Sponsor of trial*:**  Academic　 Industrial　 Unclear

***9. Date of study initiation (MM/DD/YYYY):*** [ ]

***10. Duration of study follow-up:*** Median [ ], Range [ ] to [ ]

***11. Total study duration:*** [ ] years

***12.* Method of data analysis:**  Intention to treat  Per protocol  Unclear

**Participants**

***1. Total number of study participants:***  [ ]

***2. Number of patients excluded from analysis*:**  [ ]

***3. Settings:***

**Single/multicenter**

Single center  Two center  Multicenter study  Unclear

**Inpatient/Outpatient**

Inpatient  Outpatient  Inpatient and Outpatient  Unclear

***4. Age (all allocated patients’ data are given preference)***

All allocated patients  Completer’s sample  No data

**Total** Range [ ] to [ ], Mean [ ], SD [ ]

**Zoledronate** Range [ ] to [ ], Mean [ ], SD [ ]

**Denosumab** Range [ ] to [ ], Mean [ ], SD [ ]

**Pamidronate** Range [ ] to [ ], Mean [ ], SD [ ]

**Other ( )** Range [ ] to [ ], Mean [ ], SD [ ]

**Placebo** Range [ ] to [ ], Mean [ ], SD [ ]

***5. Sex (all allocated patients’ data are given preference)***

**All allocated patients  Completer’s sample  No data**

**Total** Male [ ], Female [ ], Total [ ]

**Zoledronate** Male [ ], Female [ ], Total [ ]

**Denosumab** Male [ ], Female [ ], Total [ ]

**Pamidronate** Male [ ], Female [ ], Total [ ]

**Other ( )** Male [ ], Female [ ], Total [ ]

**Placebo** Male [ ], Female [ ], Total [ ]

***6. Performance status (ECOG):***   0  1  2  3

***7. Method of diagnosing renal cell carcinoma (RCC):***

It is clearly stated that diagnosis is histologically confirmed  Unclear

***8. Histological subtype of RCC in eligible patients:***

Clear cell RCC  Non-clear cell RCC  Both  Others [ ]  Unclear

***9. Ethnicity* [ ]**

**Intervention**

***1. Number of randomized arms:*** [ ]

***2. Number of intervention arms:*** [ ]

***3. Number of control arms:*** [ ]

***4. Number of patients randomized for intervention arm:***[ ]

***5. Number of patients randomized for control arm:*** [ ]

**<Intervention Details>**

**Arm 1**

**1. Bisphosphonate**  Zoledronate  Denosumab  Pamidronate  Other [ ]

**2. Dosage**

**A. Intended dosage** Range [ ] to [ ] mg  Unclear

**B. Prescribed dosage** Mean [ ], SD [ ] mg  Unclear

**3. Dosing schedule**

Fixed:  Everyday [ ] times/week  [ ] times/month  Other schedule [ ]

Flexible

Unclear

**4. Total duration of therapy**

[ ] days  [ ] months  [ ] years

**5. Combination therapy**

Observation (No treatment)

Irradiation for bone lesion [ ] Gy ×　[ ] fr; Total [ ] Gy

Surgery

For primary lesion:  Total nephrectomy  Partial nephrectomy  Unclear

For bone lesion:  Complete resection  Palliative surgery  Unclear

Drug therapy

| **Drug** | **Dose** | **Number of administered doses** | **Total duration** |
| --- | --- | --- | --- |
|  |  |  |  |
|  |  |  |  |
|  |  |  |  |
|  |  |  |  |

**Arm 2**

**1. Bisphosphonate**  Zoledronate  Denosumab  Pamidronate  Other [ ]

**2. Dosage**

**A. Intended dosage** Range [ ] to [ ] mg  Unclear

**B. Prescribed dosage** Mean [ ], SD [ ] mg  Unclear

**3. Dosing schedule**

Fixed:  Everyday [ ] times/week  [ ] times/month  Other schedule [ ]

Flexible

Unclear

**4. Total duration of therapy**

[ ] days  [ ] months  [ ] years

**5. Combination therapy**

Observation (No treatment)

Irradiation for bone lesion [ ] Gy ×　[ ] fr; Total [ ] Gy

Surgery

For primary lesion:  Total nephrectomy  Partial nephrectomy  Unclear

For bone lesion:  Complete resection  Palliative surgery  Unclear

Drug therapy

| **Drug** | **Dose** | **Number of administered doses** | **Total duration** |
| --- | --- | --- | --- |
|  |  |  |  |
|  |  |  |  |
|  |  |  |  |
|  |  |  |  |

**<Treatment of control group>**

**1. Treatment regimen**

Observation (No treatment)

Irradiation for bone lesion [ ] Gy ×　[ ] fr; Total [ ] Gy

Surgery for bone lesion  Complete resection  Palliative surgery  Unclear

Drug therapy

| **Drug** | **Dose** | **Number of administered doses** | **Total duration** |
| --- | --- | --- | --- |
|  |  |  |  |
|  |  |  |  |
|  |  |  |  |
|  |  |  |  |

**2. Placebo use**

Yes  No

**Outcome**

***Primary outcomes***

**Skeletal-related event (SRE)**

Definition: SRE is defined as pathologic fracture, spinal cord compression, hypercalcemia, radiation therapy, or bone surgery. We will use the definition of SRE that the authors of the original study use in each trial.

| **Intervention** | | **Control** |
| --- | --- | --- |
| **Arm 1** | **Arm 2** |  |
| ***BASELINE*: Defined as the time of [ ]** | | |
| **No. of pts [ ]** | **No. of pts [ ]** | **No. of pts [ ]** |
| ***Mid-term evaluation*: at [ ] week  month  year** | | |
| **RR [ ]**  **SE [ ]**  **95% CI [ ] to [ ]**  ***P*-value [ ]**  **No. of pts assessed [ ]** | **RR [ ]**  **SE [ ]**  **95% CI [ ] to [ ]**  ***P*-value [ ]**  **No. of pts assessed [ ]** | **RR [ ]**  **SE [ ]**  **95% CI [ ] to [ ]**  ***P*-value [ ]**  **No. of pts assessed [ ]** |
| ***End of treatment evaluation*: at [ ] week  month  year** | | |
| **RR [ ]**  **SE [ ]**  **95% CI [ ] to [ ]**  ***P*-value [ ]**  **No. of pts assessed [ ]** | **RR [ ]**  **SE [ ]**  **95% CI [ ] to [ ]**  ***P*-value [ ]**  **No. of pts assessed [ ]** | **RR [ ]**  **SE [ ]**  **95% CI [ ] to [ ]**  ***P*-value [ ]**  **No. of pts assessed [ ]** |
| ***End of follow-up evaluation*: at [ ] week  month  year**  **If the data at the original study endpoint are not mentioned so far, fill in the blanks here.** | | |
| **RR [ ]**  **SE [ ]**  **95% CI [ ] to [ ]**  ***P*-value [ ]**  **No. of pts assessed [ ]** | **RR [ ]**  **SE [ ]**  **95% CI [ ] to [ ]**  ***P*-value [ ]**  **No. of pts assessed [ ]** | **RR [ ]**  **SE [ ]**  **95% CI [ ] to [ ]**  ***P*-value [ ]**  **No. of pts assessed [ ]** |

**Severe adverse event (AE)**

Definition: AEs are defined as any unfavorable and unintended signs (including abnormal laboratory findings), symptom, or disease temporally associated with the study treatment. We will use the National Cancer Institute’s Common Terminology Criteria for Adverse Events (CTCAE), Version 4.0. We will accept any definitions that may be considered similar to this version. Severe AEs are classified as ≥grade 3.

AE grading used in this trial is  CTCAE  Others

|  | **Intervention (N)** | | | **Control (N)** |
| --- | --- | --- | --- | --- |
|  | **Arm 1** | **Arm 2** | |  |
| Osteonecrosis of the jaw  Total  Grade 3/4/5 | **[ ]**  **[ ] [ ] [ ]** | **[ ]**  **[ ] [ ] [ ]** | | **[ ]**  **[ ] [ ] [ ]** |
| Hypocalcemia  Total  Grade 3/4/5 | **[ ]**  **[ ] [ ] [ ]** | **[ ]**  **[ ] [ ] [ ]** | | **[ ]**  **[ ] [ ] [ ]** |
| Renal dysfunction  Total  Grade 3/4/5 | **[ ]**  **[ ] [ ] [ ]** | **[ ]**  **[ ] [ ] [ ]** | | **[ ]**  **[ ] [ ] [ ]** |
| [ ]  Total  Grade 3/4/5 | **[ ]**  **[ ] [ ] [ ]** | **[ ]**  **[ ] [ ] [ ]** | | **[ ]**  **[ ] [ ] [ ]** |
| [ ]  Total  Grade 3/4/5 | **[ ]**  **[ ] [ ] [ ]** | **[ ]**  **[ ] [ ] [ ]** | | **[ ]**  **[ ] [ ] [ ]** |
| [ ]  Total  Grade 3/4/5 | **[ ]**  **[ ] [ ] [ ]** | **[ ]**  **[ ] [ ] [ ]** | | **[ ]**  **[ ] [ ] [ ]** |
| [ ]  Total  Grade 3/4/5 | **[ ]**  **[ ] [ ] [ ]** | **[ ]**  **[ ] [ ] [ ]** | | **[ ]**  **[ ] [ ] [ ]** |
| [ ]  Total  Grade 3/4/5 | **[ ]**  **[ ] [ ] [ ]** | **[ ]**  **[ ] [ ] [ ]** | | **[ ]**  **[ ] [ ] [ ]** |
| [ ]  Total  Grade 3/4/5 | **[ ]**  **[ ] [ ] [ ]** | **[ ]**  **[ ] [ ] [ ]** | | **[ ]**  **[ ] [ ] [ ]** |
|  |  | |  | |

***Secondary Outcome***

**Time to the first SRE**

Definition: Time to the first SRE is measured as the duration from the beginning of treatment to the time of the first SRE.

| **Intervention** | | **Control** |
| --- | --- | --- |
| **Arm 1** | **Arm 2** |  |
| **No. of pts [ ]** | **No. of pts [ ]** | **No. of pts [ ]** |
| **Mean [ ] months**  **SD [ ]**  **Range [ ] to [ ] months**  **No. of pts assessed [ ]** | **Mean [ ] months**  **SD [ ]**  **Range [ ] to [ ] months**  **No. of pts assessed [ ]** | **Mean [ ] months**  **SD [ ]**  **Range [ ] to [ ] months**  **No. of pts assessed [ ]** |

**Skeletal morbidity rate (SMR)**

Definition: SMR is defined as the ratio of the number of SREs for each subject divided by the subject’s time at risk in years. For example, if a study follows 1000 patients for 1 year and among those 1000 patients, 350 SREs occur, the SMR value would be 0.35 SREs/year. If multiple events occur within a year, these values are included within the ratio.

| **Intervention** | | **Control** |
| --- | --- | --- |
| **Arm 1** | **Arm 2** |  |
| ***BASELINE*: Defined as the time of [ ]** | | |
| **No. of pts [ ]** | **No. of pts [ ]** | **No. of pts [ ]** |
| ***1-year follow-up*** | | |
| **HR [ ]**  **SE [ ]**  **CI [ ] to [ ]**  ***P*-value [ ]**  **No. of deaths [ ]**  **No. of pts assessed [ ]** | **HR [ ]**  **SE [ ]**  **CI [ ] to [ ]**  ***P*-value [ ]**  **No. of deaths [ ]**  **No. of pts assessed [ ]** | **HR [ ]**  **SE [ ]**  **CI [ ] to [ ]**  ***P*-value [ ]**  **No. of deaths [ ]**  **No. of pts assessed [ ]** |
| ***2-year follow-up*** | | |
| **HR [ ]**  **SE [ ]**  **CI [ ] to [ ]**  ***P*-value [ ]**  **No. of deaths [ ]**  **No. of pts assessed [ ]** | **HR [ ]**  **SE [ ]**  **CI [ ] to [ ]**  ***P*-value [ ]**  **No. of deaths [ ]**  **No. of pts assessed [ ]** | **HR [ ]**  **SE [ ]**  **CI [ ] to [ ]**  ***P*-value [ ]**  **No. of deaths [ ]**  **No. of pts assessed [ ]** |
| ***3-year follow-up*** | | |
| **HR [ ]**  **SE [ ]**  **CI [ ] to [ ]**  ***P*-value [ ]**  **No. of deaths [ ]**  **No. of pts assessed [ ]** | **HR [ ]**  **SE [ ]**  **CI [ ] to [ ]**  ***P*-value [ ]**  **No. of deaths [ ]**  **No. of pts assessed [ ]** | **HR [ ]**  **SE [ ]**  **CI [ ] to [ ]**  ***P*-value [ ]**  **No. of deaths [ ]**  **No. of pts assessed [ ]** |
| ***4-year follow-up*** | | |
| **HR [ ]**  **SE [ ]**  **CI [ ] to [ ]**  ***P*-value [ ]**  **No. of deaths [ ]**  **No. of pts assessed [ ]** | **HR [ ]**  **SE [ ]**  **CI [ ] to [ ]**  ***P*-value [ ]**  **No. of deaths [ ]**  **No. of pts assessed [ ]** | **HR [ ]**  **SE [ ]**  **CI [ ] to [ ]**  ***P*-value [ ]**  **No. of deaths [ ]**  **No. of pts assessed [ ]** |
| ***5-year follow-up*** | | |
| **HR [ ]**  **SE [ ]**  **CI [ ] to [ ]**  ***P*-value [ ]**  **No. of deaths [ ]**  **No. of pts assessed [ ]** | **HR [ ]**  **SE [ ]**  **CI [ ] to [ ]**  ***P*-value [ ]**  **No. of death [ ]**  **No. of pts assessed [ ]** | **HR [ ]**  **SE [ ]**  **CI [ ] to [ ]**  ***P*-value [ ]**  **No. of death [ ]**  **No. of pts assessed [ ]** |
| ***END OF FOLLOW-UP*: Defined as the time of [ ]**  **If the data at original study endpoint are not mentioned so far, fill-in the blanks here.** | | |
| **HR [ ]**  **SE [ ]**  **CI [ ] to [ ]**  ***P*-value [ ]**  **No. of death [ ]**  **No. of pts assessed [ ]** | **HR [ ]**  **SE [ ]**  **CI [ ] to [ ]**  ***P*-value [ ]**  **No. of deaths [ ]**  **No. of pts assessed [ ]** | **HR [ ]**  **SE [ ]**  **CI [ ] to [ ]**  ***P*-value [ ]**  **No. of deaths [ ]**  **No. of pts assessed [ ]** |

**Non-severe adverse event (AE)**

Definition: AEs are defined as mentioned above. Non-severe AEs are classified as ≤grade 2.

AE grading used in this trial is  CTCAE  Others

|  | **Intervention (N)** | | | **Control (N)** |
| --- | --- | --- | --- | --- |
|  | **Arm 1** | **Arm 2** | |  |
| Osteonecrosis of the jaw  Total  Grade 1/2 | **[ ]**  **[ ] [ ]** | **[ ]**  **[ ] [ ]** | | **[ ]**  **[ ] [ ]** |
| Hypocalcemia  Total  Grade 1/2 | **[ ]**  **[ ] [ ]** | **[ ]**  **[ ] [ ]** | | **[ ]**  **[ ] [ ]** |
| Renal dysfunction  Total  Grade 1/2 | **[ ]**  **[ ] [ ]** | **[ ]**  **[ ] [ ]** | | **[ ]**  **[ ] [ ]** |
| [ ]  Total  Grade 1/2 | **[ ]**  **[ ] [ ]** | **[ ]**  **[ ] [ ]** | | **[ ]**  **[ ] [ ]** |
| [ ]  Total  Grade 1/2 | **[ ]**  **[ ] [ ]** | **[ ]**  **[ ] [ ]** | | **[ ]**  **[ ] [ ]** |
| [ ]  Total  Grade 1/2 | **[ ]**  **[ ] [ ]** | **[ ]**  **[ ] [ ]** | | **[ ]**  **[ ] [ ]** |
| [ ]  Total  Grade 1/2 | **[ ]**  **[ ] [ ]** | **[ ]**  **[ ] [ ]** | | **[ ]**  **[ ] [ ]** |
| [ ]  Total  Grade 1/2 | **[ ]**  **[ ] [ ]** | **[ ]**  **[ ] [ ]** | | **[ ]**  **[ ] [ ]** |
| [ ]  Total  Grade 1/2 | **[ ]**  **[ ] [ ]** | **[ ]**  **[ ] [ ]** | | **[ ]**  **[ ] [ ]** |
|  |  | |  | |

**Overall Survival (OS)**

Definition: Time from enrollment in the study until death. If a trial reports OS from any time point after disease diagnosis, and not from randomization, we will not exclude it.

| **Intervention** | | **Control** |
| --- | --- | --- |
| **Arm 1** | **Arm 2** |  |
| ***BASELINE*: Defined as the time of [ ]** | | |
| **No. of pts [ ]** | **No. of pts [ ]** | **No. of pts [ ]** |
| ***1-year follow-up*** | | |
| **HR [ ]**  **SE [ ]**  **CI [ ] to [ ]**  ***P*-value [ ]**  **No. of deaths [ ]**  **No. of pts assessed [ ]** | **HR [ ]**  **SE [ ]**  **CI [ ] to [ ]**  ***P*-value [ ]**  **No. of deaths [ ]**  **No. of pts assessed [ ]** | **HR [ ]**  **SE [ ]**  **CI [ ] to [ ]**  ***P*-value [ ]**  **No. of deaths [ ]**  **No. of pts assessed [ ]** |
| ***2-year follow-up*** | | |
| **HR [ ]**  **SE [ ]**  **CI [ ] to [ ]**  ***P*-value [ ]**  **No. of deaths [ ]**  **No. of pts assessed [ ]** | **HR [ ]**  **SE [ ]**  **CI [ ] to [ ]**  ***P*-value [ ]**  **No. of deaths [ ]**  **No. of pts assessed [ ]** | **HR [ ]**  **SE [ ]**  **CI [ ] to [ ]**  ***P*-value [ ]**  **No. of deaths [ ]**  **No. of pts assessed [ ]** |
| ***3-year follow-up*** | | |
| **HR [ ]**  **SE [ ]**  **CI [ ] to [ ]**  ***P*-value [ ]**  **No. of deaths [ ]**  **No. of pts assessed [ ]** | **HR [ ]**  **SE [ ]**  **CI [ ] to [ ]**  ***P*-value [ ]**  **No. of deaths [ ]**  **No. of pts assessed [ ]** | **HR [ ]**  **SE [ ]**  **CI [ ] to [ ]**  ***P*-value [ ]**  **No. of deaths [ ]**  **No. of pts assessed [ ]** |
| ***4-year follow-up*** | | |
| **HR [ ]**  **SE [ ]**  **CI [ ] to [ ]**  ***P*-value [ ]**  **No. of deaths [ ]**  **No. of pts assessed [ ]** | **HR [ ]**  **SE [ ]**  **CI [ ] to [ ]**  ***P*-value [ ]**  **No. of deaths [ ]**  **No. of pts assessed [ ]** | **HR [ ]**  **SE [ ]**  **CI [ ] to [ ]**  ***P*-value [ ]**  **No. of deaths [ ]**  **No. of pts assessed [ ]** |
| ***5-year follow-up*** | | |
| **HR [ ]**  **SE [ ]**  **CI [ ] to [ ]**  ***P*-value [ ]**  **No. of deaths [ ]**  **No. of pts assessed [ ]** | **HR [ ]**  **SE [ ]**  **CI [ ] to [ ]**  ***P*-value [ ]**  **No. of deaths [ ]**  **No. of pts assessed [ ]** | **HR [ ]**  **SE [ ]**  **CI [ ] to [ ]**  ***P*-value [ ]**  **No. of deaths [ ]**  **No. of pts assessed [ ]** |
| ***END OF FOLLOW-UP*: Defined as the time of [ ]**  **If the data at original study endpoint are not mentioned so far, fill-in the blanks here.** | | |
| **HR [ ]**  **SE [ ]**  **CI [ ] to [ ]**  ***P*-value [ ]**  **No. of deaths [ ]**  **No. of pts assessed [ ]** | **HR [ ]**  **SE [ ]**  **CI [ ] to [ ]**  ***P*-value [ ]**  **No. of deaths [ ]**  **No. of pts assessed [ ]** | **HR [ ]**  **SE [ ]**  **CI [ ] to [ ]**  ***P*-value [ ]**  **No. of deaths [ ]**  **No. of pts assessed [ ]** |

**Progression-free Survival (PFS)**

Definition: Defined as the time from enrollment in the study until progression or death. If a trial reports PFS from any time point after the disease diagnosis, and not from randomization, we will not exclude it. We will use the definition of progression that the authors of the original study used in each trial.

| **Intervention** | | **Control** |
| --- | --- | --- |
| **Arm 1** | **Arm 2** |  |
| ***BASELINE*: Defined as the time of [ ]** | | |
| **No. of pts [ ]** | **No. of pts [ ]** | **No. of pts [ ]** |
| ***1-year follow-up*** | | |
| **HR [ ]**  **SE [ ]**  **CI [ ] to [ ]**  ***P*-value [ ]**  **No. of progression**  **[ ]**  **No. of pts assessed [ ]** | **HR [ ]**  **SE [ ]**  **CI [ ] to [ ]**  ***P*-value [ ]**  **No. of progression**  **[ ]**  **No. of pts assessed [ ]** | **HR [ ]**  **SE [ ]**  **CI [ ] to [ ]**  ***P*-value [ ]**  **No. of progression**  **[ ]**  **No. of pts assessed [ ]** |
| ***2-year follow-up*** | | |
| **HR [ ]**  **SE [ ]**  **CI [ ] to [ ]**  ***P*-value [ ]**  **No. of progression**  **[ ]**  **No. of pts assessed [ ]** | **HR [ ]**  **SE [ ]**  **CI [ ] to [ ]**  ***P*-value [ ]**  **No. of progression**  **[ ]**  **No. of pts assessed [ ]** | **HR [ ]**  **SE [ ]**  **CI [ ] to [ ]**  ***P*-value [ ]**  **No. of progression**  **[ ]**  **No. of pts assessed [ ]** |
| ***3-year follow-up*** | | |
| **HR [ ]**  **SE [ ]**  **CI [ ] to [ ]**  ***P*-value [ ]**  **No. of progression**  **[ ]**  **No. of pts assessed [ ]** | **HR [ ]**  **SE [ ]**  **CI [ ] to [ ]**  ***P*-value [ ]**  **No. of progression**  **[ ]**  **No. of pts assessed [ ]** | **HR [ ]**  **SE [ ]**  **CI [ ] to [ ]**  ***P*-value [ ]**  **No. of progression**  **[ ]**  **No. of pts assessed [ ]** |
| ***4-year follow-up*** | | |
| **HR [ ]**  **SE [ ]**  **CI [ ] to [ ]**  ***P*-value [ ]**  **No. of progression**  **[ ]**  **No. of pts assessed [ ]** | **HR [ ]**  **SE [ ]**  **CI [ ] to [ ]**  ***P*-value [ ]**  **No. of progression**  **[ ]**  **No. of pts assessed [ ]** | **HR [ ]**  **SE [ ]**  **CI [ ] to [ ]**  ***P*-value [ ]**  **No. of progression**  **[ ]**  **No. of pts assessed [ ]** |
| ***5-year follow-up*** | | |
| **HR [ ]**  **SE [ ]**  **CI [ ] to [ ]**  ***P*-value [ ]**  **No. of progression**  **[ ]**  **No. of pts assessed [ ]** | **HR [ ]**  **SE [ ]**  **CI [ ] to [ ]**  ***P*-value [ ]**  **No. of progression**  **[ ]**  **No. of pts assessed [ ]** | **HR [ ]**  **SE [ ]**  **CI [ ] to [ ]**  ***P*-value [ ]**  **No. of progression**  **[ ]**  **No. of pts assessed [ ]** |
| ***END OF FOLLOW-UP*: Defined as the time of [ ]**  **If the data at original study endpoint are not mentioned so far, fill-in the blanks here.** | | |
| **HR [ ]**  **SE [ ]**  **CI [ ] to [ ]**  ***P*-value [ ]**  **No. of death**  **[ ]**  **No. of pts assessed [ ]** | **HR [ ]**  **SE [ ]**  **CI [ ] to [ ]**  ***P*-value [ ]**  **No. of death**  **[ ]**  **No. of pts assessed [ ]** | **HR [ ]**  **SE [ ]**  **CI [ ] to [ ]**  ***P*-value [ ]**  **No. of death**  **[ ]**  **No. of pts assessed [ ]** |

**Health-related Quality of Life (QOL)**

Definition: All emotional, social, and physical aspects a of participant’s life that are affected by their disease and study treatment; QOL is evaluated by validated questionnaires and scaling systems.

Scale:  SF-36  EQ-5D  Other [ ]

| **Intervention** | | **Control** |
| --- | --- | --- |
| **Arm 1** | **Arm 2** |  |
| ***BASELINE*: Defined as the time of [ ]** | | |
| **No. of pts [ ]** | **No. of pts [ ]** | **No. of pts [ ]** |
| ***1st follow-up*: at [ ] week  month  year** | | |
| **Mean [ ]**  **SD [ ]**  **Range [ ] to [ ]**  **No. of pts assessed [ ]** | **Mean [ ]**  **SD [ ]**  **Range [ ] to [ ]**  **No. of pts assessed [ ]** | **Mean [ ]**  **SD [ ]**  **Range [ ] to [ ]**  **No. of pts assessed [ ]** |
| ***2nd follow-up*: at [ ] week  month  year** | | |
| **Mean [ ]**  **SD [ ]**  **Range [ ] to [ ]**  **No. of pts assessed [ ]** | **Mean [ ]**  **SD [ ]**  **Range [ ] to [ ]**  **No. of pts assessed [ ]** | **Mean [ ]**  **SD [ ]**  **Range [ ] to [ ]**  **No. of pts assessed [ ]** |
| ***3rd follow-up*: at [ ] week  month  year** | | |
| **Mean [ ]**  **SD [ ]**  **Range [ ] to [ ]**  **No. of pts assessed [ ]** | **Mean [ ]**  **SD [ ]**  **Range [ ] to [ ]**  **No. of pts assessed [ ]** | **Mean [ ]**  **SD [ ]**  **Range [ ] to [ ]**  **No. of pts assessed [ ]** |
| ***[ ]th follow-up*: at [ ] week  month  year** | | |
| **Mean [ ]**  **SD [ ]**  **Range [ ] to [ ]**  **No. of pts assessed [ ]** | **Mean [ ]**  **SD [ ]**  **Range [ ] to [ ]**  **No. of pts assessed [ ]** | **Mean [ ]**  **SD [ ]**  **Range [ ] to [ ]**  **No. of pts assessed [ ]** |
| ***End of follow-up evaluation*: at [ ] week  month  year**  **If the data at original study endpoint are not mentioned so far, fill-in the blanks here.** | | |
| **Mean [ ]**  **SD [ ]**  **Range [ ] to [ ]**  **No. of pts assessed [ ]** | **Mean [ ]**  **SD [ ]**  **Range [ ] to [ ]**  **No. of pts assessed [ ]** | **Mean [ ]**  **SD [ ]**  **Range [ ] to [ ]**  **No. of pts assessed [ ]** |

**Cost effectiveness (CE)**

Definition: CE is measured as the incremental cost-effective ratio (ICER)

Unit of ICER is

Dollars/life-year gained (LYG)

Dollars/quality-adjusted life year (QALY)

Other [ ]

| **Intervention** | | **Control** |
| --- | --- | --- |
| **Arm 1** | **Arm 2** |  |
| **No. of pts [ ]** | **No. of pts [ ]** | **No. of pts [ ]** |
| **Mean [ ]**  **SD [ ]**  **Range [ ] to [ ]**  **No. of pts assessed [ ]** | **Mean [ ]**  **SD [ ]**  **Range [ ] to [ ]**  **No. of pts assessed [ ]** | **Mean [ ]**  **SD [ ]**  **Range [ ] to [ ]**  **No. of pts assessed [ ]** |

**Number of dropouts and loss to follow-up**

|  | **Intervention** | | **Control** |
| --- | --- | --- | --- |
|  | **Arm 1** | **Arm 2** |  |
| **Total number of allocated pts** | **[ ]** | **[ ]** | **[ ]** |
| **Total dropouts** | **[ ]** | **[ ]** | **[ ]** |
| Due to inefficiency | **[ ]** | **[ ]** | **[ ]** |
| **Loss to follow-up** | **[ ]** | **[ ]** | **[ ]** |
| Due to side effect | **[ ]** | **[ ]** | **[ ]** |
| Due to other reasons | **[ ]** | **[ ]** | **[ ]** |
| Reasons unclear | **[ ]** | **[ ]** | **[ ]** |
