# Supplementary material for: Comparative efficacy and safety of bone-modifying agents for the treatment of bone metastases in patients with advanced renal cell carcinoma: a systematic review and meta-analysis
Source: Oncotarget. 2017 Aug 18;8(40):68890–8. doi: 10.18632/oncotarget.20323 (PMC5620305; doi:10.18632/oncotarget.20323)
Supplement: Supplementary file 4 [file oncotarget-08-68890-s004.docx]

**Appendix 7. Criteria for judging risk of bias in the “risk of bias” assessment tool**

| **RANDOM SEQUENCE GENERATION**  **Selection bias (biased allocation to interventions) due to inadequate generation of a randomized sequence** | |
| --- | --- |
| **Criteria for judgment of “low risk” of bias** | **The investigators describe a random component in the sequence-generation process. Some examples are as follows :**   - **Referring to a random number table** - **Using a computer random number generator** - **Coin tossing** - **Shuffling cards or envelopes** - **Throwing dice** - **Drawing of lots** - **Minimization***     ***Minimization may be implemented without a random element, and this is considered to be equivalent to randomization.** |
| **Criteria for judgment of “high risk” of bias** | **The investigators describe a non-random component in the sequence-generation process. Usually, the description would involve a systematic, non-random approach. Some examples are as follows:**   - **Sequence generated by odd or even date of birth** - **Sequence generated by a rule based on date (or day) of admission** - **Sequence generated by a rule based on hospital or clinic record number**     **Other non-random approaches are used much less frequently than the systematic approaches mentioned above and tend to be obvious. They usually involve judgment or some method of non-random categorization of participants. Some examples are as follows:**   - **Allocation by judgment by the clinician** - **Allocation by preference of the participant** - **Allocation based on the results of a laboratory test or a series of tests** - **Allocation by availability of the intervention** |
| **Criteria for judgment of “unclear risk” of bias** | **Insufficient information about the sequence-generation process to permit judgment of “low risk” or “high risk”** |
| **ALLOCATION CONCEALMENT**  **Selection bias (biased allocation to interventions) due to inadequate concealment of allocations prior to assignment** | |
| **Criteria for judgment of “low risk” of bias** | **Participants and investigators enrolling participants could not foresee assignment because one of the following, or an equivalent method, was used to conceal allocation:**   - **Central allocation (including telephonic, web-based, and pharmacy-controlled randomization)** - **Sequentially numbered drug containers of identical appearance** - **Sequentially numbered, opaque, sealed envelopes** |
| **Criteria for judgment of “high risk” of bias** | **Participants or investigators enrolling participants could possibly foresee assignments and thus introduced selection bias. For example, allocation based on the following:**   - **An open, random-allocation schedule (e.g., a list of random numbers)** - **Assignment envelopes used without appropriate safeguards (e.g., if envelopes were unsealed, non-­opaque, or not sequentially numbered)** - **Alternation or rotation** - **Date of birth** - **Case record number** - **Any other explicitly unconcealed procedure** |
| **Criteria for judgment of “unclear risk” of bias** | **Insufficient information to permit judgment of “low risk” or “high risk.” This is usually the case if the method of concealment is not described at all or in sufficient detail to allow a definite judgment; for example, the use of assignment envelopes is described, but it remains unclear whether the envelopes were sequentially numbered, opaque, and sealed.** |
| **BLINDING OF PARTICIPANTS AND PERSONNEL**  **Performance bias due to knowledge of the allocated interventions by participants and personnel during the study** | |
| **Criteria for judgment of “low risk” of bias** | **Any one of the following:**   - **No blinding or incomplete blinding, but the review authors believe that the outcome is not likely to be influenced by the lack of blinding** - **Blinding of participants and key study personnel ensured, and unlikely that the blinding could have been broken** |
| **Criteria for judgment of “low risk” of bias** | **Any one of the following:**   - **No blinding or incomplete blinding, and the outcome is likely to be influenced by the lack of blinding** - **Blinding of key study participants and personnel attempted, but likely that the blinding could have been undone, and the outcome is likely to be influenced by the lack of blinding** |
| **Criteria for judgment of “unclear risk” of bias** | **Any one of the following:**   - **Insufficient information to permit judgment of “low risk” or “high risk”** - **The study did not address this outcome** |
| **BLINDING OF OUTCOME ASSESSMENT**  **Detection bias due to knowledge of the allocated interventions among outcome assessors** | |
| **Criteria for judgment of “low risk” of bias** | **Any one of the following:**   - **No blinding of outcome assessment, but the review authors believe that the outcome measurement is not likely to be influenced by the lack of blinding** - **Blinding of outcome assessment ensured, and unlikely that the blinding could have been broken** |
| **Criteria for judgment of “high risk” of bias** | **Any one of the following:**   - **No blinding of outcome assessment, and the outcome measurement is likely to be influenced by the lack of blinding** - **Blinding of outcome assessment, but likely that the blinding could have been broken, and the outcome measurement is likely to be influenced by the lack of blinding** |
| **Criteria for judgment of “unclear risk” of bias** | **Any one of the following:**   - **Insufficient information to permit judgment of “low risk” or “high risk”** - **The study did not address this outcome** |
| **INCOMPLETE OUTCOME DATA**  **Attrition bias due to amount, nature, or handling of incomplete outcome data** | |
| **Criteria for judgment of “low risk” of bias** | **Any one of the following:**   - **No missing outcome data** - **Reasons for missing outcome data are unlikely to be related to true outcome (for survival data, censoring unlikely to introduce bias)** - **Missing outcome data are balanced in numbers across intervention groups, with similar reasons for missing data across groups** - **For dichotomous outcome data, the proportion of missing outcomes compared with observed event risk is not sufficient for a clinically relevant impact on the intervention-effect estimate** - **For continuous outcome data, plausible effect size (difference in means or standardized difference in means) among missing outcomes not sufficient for a clinically relevant impact on observed effect size;** - **Missing data have been imputed using appropriate methods** |
| **Criteria for judgment of “high risk” of bias** | **Any one of the following:**   - **Reason for missing outcome data are likely to be related to true outcome, with either imbalance in numbers or reasons for missing data across intervention groups** - **For dichotomous outcome data, the proportion of missing outcomes compared with observed event risk is enough to induce clinically relevant bias in intervention-effect estimate** - **For continuous outcome data, plausible effect size (difference in means or standardized difference in means) among missing outcomes is enough to induce clinically relevant bias in observed-effect size** - **“As-treated” analysis performed with substantial departure of the intervention received from that assigned at randomization** - **Potentially inappropriate application of simple imputation** |
| **Criteria for judgment of “unclear risk” of bias** | **Any one of the following:**   - **Insufficient reporting of attrition/exclusions to permit judgment of “low risk” or “high risk” (e.g., number of randomized cases not stated, no reasons for missing data provided)** - **The study did not address this outcome** |
| **SELECTIVE REPORTING**  **Reporting bias due to selective outcome reporting** | |
| **Criteria for judgment of “low risk” of bias** | **Any of the following:**   - **The study protocol is available and all of the pre-specified (primary and secondary) outcomes that are of interest in the review have been reported in the pre-specified way** - **The study protocol is not available but it is clear that the published reports include all expected outcomes, including pre-specified outcomes (convincing text of this nature may be uncommon)** |
| **Criteria for judgment of “high risk” of bias** | **Any one of the following:**   - **Not all the pre-specified primary outcomes have been reported** - **One or more primary outcomes are reported using measurements, analysis methods, or subsets of data (e.g., subscales) that were not pre-specified** - **One or more reported primary outcomes were not pre-specified (unless clear justification for their reporting is provided, such as an unexpected adverse effect)** - **One or more outcomes of interest in the review are reported incompletely, and therefore, they cannot be entered in a meta-analysis** - **The study fails to include results for a key outcome that would be expected to have been reported for such a study** |
| **Criteria for judgment of “unclear risk” of bias** | **Insufficient information to permit judgment of “low risk” or “high risk.” It is likely that the majority of studies will fall into this category.** |
| **OTHER BIAS**  **Bias due to problems not covered elsewhere in the table** | |
| **Criteria for judgment of “low risk” of bias** | **The study appears to be free of other sources of bias** |
| **Criteria for judgment of “high risk” of bias** | **There is at least one important risk of bias. For example, the study includes one of the following:**   - **A potential source of bias related to blinding of data analysis** - **Pre-randomization administration of an intervention, inappropriate administration of an intervention, or co-intervention** - **Baseline imbalance** - **Early stopping or supplements for the dropouts by providing additional recruits, or recruits additional participants from a subgroup showing more or less benefit** - **Deviation from the study protocol in a way that does not reflect clinical practice** - **A potential source of bias related to differential diagnostic activity** - **A potential source of bias related to pharmaceutical funding and/or support** |
| **Criteria for judgment of “unclear risk” of bias** | **There may be a risk of bias, but there is one of the following:**   - **Insufficient information to assess whether an important risk of bias exists** - **Insufficient rationale or evidence that an identified problem will introduce bias** |
